# Honeycomb-like biomimetic scaffold by functionalized antibacterial hydrogel and biodegradable porous Mg alloy for osteochondral regeneration

**DOI:** 10.3389/fbioe.2024.1417742

**Published:** 2024-07-12

**Authors:** Yongqiang Zhang, Qiangsheng Dong, Xiao Zhao, Yuzhi Sun, Xin Lin, Xin Zhang, Tianming Wang, Tianxiao Yang, Xiao Jiang, Jiaxiang Li, Zhicheng Cao, Tingwen Cai, Wanshun Liu, Hongjing Zhang, Jing Bai, Qingqiang Yao

**Affiliations:** ^1^ Department of Orthopedic Surgery, Institute of Digital Medicine, Nanjing First Hospital, Nanjing Medical University, Nanjing, China; ^2^ Key Lab of Additive Manufacturing Technology, Institute of Digital Medicine, Nanjing Medical University, Nanjing, China; ^3^ Research Center of Digital Medicine and 3D Printing Technology of Jiangsu Province, Nanjing, China; ^4^ School of Materials Science and Engineering, Jiangsu Key Laboratory of Advanced Structural Materials and Application Technology, Nanjing Institute of Technology, Nanjing, China; ^5^ Department of Laboratory Medicine, Nanjing First Hospital, Nanjing Medical University, Nanjing, Jiangsu, China; ^6^ School of Materials Science and Engineering, Southeast University, Nanjing, Jiangsu, China; ^7^ Institute of Medical Devices (Suzhou), Southeast University, Suzhou, China

**Keywords:** osteochondral defects, biomimetic scaffold, honeycomb, magnesium alloy, sodium alginate hydrogel, zinc ions

## Abstract

**Introduction:** Osteochondral repair poses a significant challenge due to its unique pathological mechanisms and complex repair processes, particularly in bacterial tissue conditions resulting from open injuries, infections, and surgical contamination. This study introduces a biomimetic honeycomb-like scaffold (Zn-AlgMA@Mg) designed for osteochondral repair. The scaffold consists of a dicalcium phosphate dihydrate (DCPD)-coated porous magnesium scaffold (DCPD Mg) embedded within a dual crosslinked sodium alginate hydrogel (Zn-AlgMA). This combination aims to synergistically exert antibacterial and osteochondral integrated repair properties.

**Methods:** The Zn-AlgMA@Mg scaffold was fabricated by coating porous magnesium scaffolds with DCPD and embedding them within a dual crosslinked sodium alginate hydrogel. The structural and mechanical properties of the DCPD Mg scaffold were characterized using scanning electron microscopy (SEM) and mechanical testing. The microstructural features and hydrophilicity of Zn-AlgMA were assessed. *In vitro* studies were conducted to evaluate the controlled release of magnesium and zinc ions, as well as the scaffold’s osteogenic, chondrogenic, and antibacterial properties. Proteomic analysis was performed to elucidate the mechanism of osteochondral integrated repair. *In vivo* efficacy was evaluated using a rabbit full-thickness osteochondral defect model, with micro-CT evaluation, quantitative analysis, and histological staining (hematoxylin-eosin, Safranin-O, and Masson’s trichrome).

**Results:** The DCPD Mg scaffold exhibited a uniform porous structure and superior mechanical properties. The Zn-AlgMA hydrogel displayed consistent microstructural features and enhanced hydrophilicity. The Zn-AlgMA@Mg scaffold provided controlled release of magnesium and zinc ions, promoting cell proliferation and vitality. *In vitro* studies demonstrated significant osteogenic and chondrogenic properties, as well as antibacterial efficacy. Proteomic analysis revealed the underlying mechanism of osteochondral integrated repair facilitated by the scaffold. Micro-CT evaluation and histological analysis confirmed successful osteochondral integration in the rabbit model.

**Discussion:** The biomimetic honeycomb-like scaffold (Zn-AlgMA@Mg) demonstrated promising results for osteochondral repair, effectively addressing the challenges posed by bacterial tissue conditions. The scaffold’s ability to release magnesium and zinc ions in a controlled manner contributed to its significant osteogenic, chondrogenic, and antibacterial properties. Proteomic analysis provided insights into the scaffold’s mechanism of action, supporting its potential for integrated osteochondral regeneration. The successful *in vivo* results highlight the scaffold’s efficacy, making it a promising biomaterial for future applications in osteochondral repair.

## 1 Introduction

As the incidence of joint trauma increases from sports injuries, traffic accidents and age-related osteoarthritis (OA), the prevalence of bone and cartilage injuries is rising annually (Wang et al., 2017; Allen et al., 2022). These injuries have become a major cause of disability along with joint pain and functional impairment (Safiri et al., 2020). Current clinical treatments for bone and cartilage injuries include core decompression, microfracture surgery, bone marrow stimulation, and autologous/allogenic tissue transplantation (Nazem et al., 2011). While these treatments can alleviate pain and restore some of the damaged tissue, they are limited by donor site morbidity (Minehara et al., 2023) and potential for rejection, failing to achieve complete tissue regeneration. Tissue engineering techniques offer further opportunities for ideal bone repair (Şeker et al., 2023). Biomimetic scaffolds not only provide a substrate for cell adhesion but also induce cell proliferation and differentiation. They can release bioactive substances on a degradable basis, influencing cell response and regulating the synthesis and assembly of the extracellular matrix, thereby initiating the body’s regenerative system (Shimomura et al., 2014).

Today, bee hive products (honey, pollen, propolis, and bee venom) have been developed as a non-pharmacological therapy for curing various inflammatory diseases (Almuhareb et al., 2019; Conrad et al., 2019; Hsieh et al., 2019). Among them, honey, containing a variety of bioactive components (enzymes, amino acids, proteins, flavonoids, phenolic acids, organic acids, vitamins, and minerals), has been proven to have anti-inflammatory, antibacterial, and antioxidant effects (Alvarez-Suarez et al., 2010; Li Y. et al., 2019; Yuan et al., 2020; Orhan and Deniz, 2021). Notably, the zinc ion content (0.05–2 mg/100 g) in honey is rich (Ajibola et al., 2012), offering cartilage protection and antibacterial benefits. On the other hand, the magnesium ion content in bee combs (2 mg/100 g) is also significant, promoting osteogenesis and neuromodulation (Zhao et al., 2020). Furthermore, the porous structure of the bee comb, capable of withstanding high compressive and tensile stresses, mimics the microstructure of natural bone tissue (Lu et al., 2020), providing essential porosity crucial for cell attachment and proliferation (Wang et al., 2021). Therefore, inspired by the composition, structure, and functionality of bee hives, the biomimetic construction of a honeycomb-like scaffold for integrated bone and cartilage repair holds significant importance.

Inspired by honeycomb and honey, A biomimetic tissue engineering scaffold was designed in this work, in which honey-like hydrogels were embedded in honeycomb-like magnesium alloy scaffolds. Magnesium and zinc elements were used to build the biomimetic tissue engineering scaffold, referred to the characteristics of natural honeycomb and honey. This scaffold aims to facilitate integrated repair of osteochondral defects. Magnesium, the fourth most abundant metal in the human body, is involved in hundreds of biochemical reactions. Its alloys, being biodegradable metals, not only possess excellent biocompatibility and Young’s modulus close to natural bone (Zhang et al., 2022), but also effectively reduce stress shielding and eliminate secondary surgery for the removal of the implant, widely used in bone defect repair treatments (He et al., 2023). Accordingly, the honeycomb-like magnesium alloy scaffold was coated with DCPD (dicalcium phosphate dihydrate) to slow the magnesium alloy degradation and improve the bioactivity, thereby enhancing the overall material performance. The gradual degradation of magnesium alloy releases magnesium ions, simulating the physiological action of a honeycomb. Furthermore, as regenerative medicine research advances, hydrogels, due to their structural and functional resemblance to articular cartilage and excellent biocompatibility, are considered ideal substitutes for joint cartilage (Mehta et al., 2023). Sodium alginate, a natural polysaccharide extracted from brown algae, comprising alternating units of β-D-mannuronic acid and α-L-guluronic acid, not only excels in hydrogel formation but also ensures high biocompatibility. Therefore, the “honey” part of our scaffold uses a sodium alginate hydrogel with a dual-crosslinked structure (zinc ion and photo-crosslinking). The methacrylate modification and zinc ion crosslinking significantly enhance the mechanical properties and stability of the sodium alginate hydrogel. Moreover, the gradual release of zinc ions during *in vivo* degradation simulates the physiological action of honey, aiming to continuously promote cartilage tissue growth.

Although recent years have seen an increase in studies on magnesium-based metallic scaffolds for bone defect repair and organic hydrogels for cartilage repair, research on integrated osteochondral repair using a biomimetic scaffold combining organic hydrogel and inorganic metallic scaffold is still lacking. This experiment systematically characterizes the morphology and physicochemical properties of this organic-inorganic honeycomb-like scaffold through a series of *in vitro* experiments. Its biological characteristics, including cell compatibility, antibacterial properties, and differentiation potential were evaluated by using bone marrow mesenchymal stem cells (BMSCs). Proteomic analysis further investigates the pathways and mechanisms by which the honeycomb-like scaffold affects cellular behavior. Finally, the *in vivo* osteochondral regeneration capacity of the honeycomb-like scaffold is evaluated by implanting it into a rabbit femoral osteochondral defects model.

## 2 Experimental

### 2.1 Preparation of the magnesium scaffold

Spherical NaCl particles were sintered in an electric furnace to create an open porous NaCl template. The template was then infiltrated under a pressure of 0.2 MPa in a casting process using molten Mg to fill the template. Upon solidification, a composite of Mg and NaCl was successfully synthesized. The magnesium scaffold was obtained by leaching out the NaCl template using running water. The scaffold was further etched with 4% nitric acid-ethanol solution in an ultrasonic cleaner for 30 s to achieve a clean surface.

### 2.2 Application of DCPD coating

The magnesium alloy samples were cleaned in an ultrasonic cleaner with ethanol and deionized water for 30 min each, followed by natural drying at room temperature. A 0.05M solution of DCPD powder in water was prepared, and the DCPD solution was uniformly sprayed onto the surface of the magnesium alloy at a rate of 0.2 mL/min using a flow field device. The spraying process was conducted in a controlled environment at a constant temperature of 25°C and a relative humidity of 60%. After spraying, the samples were left to stand at room temperature for 24 h, then thermally treated in an oven at 150°C for 2 h. The samples were naturally cooled to room temperature after heat treatment for subsequent characterization and testing.

### 2.3 Structural characterization

An X-ray tomography scanner was used to scan the open porous Mg scaffold. The software VG analyzed the pore size distribution, surface area, and porosity of the Mg scaffold. The microstructure and surface composition were characterized using a Scanning Electron Microscope (SEM) equipped with an Energy Dispersive X-ray Spectroscopy (EDS).

### 2.4 Compression testing

A CMT4503 universal testing machine was used for room temperature uniaxial compression testing to characterize the mechanical properties of the Mg scaffold. Tests were conducted on scaffolds with a diameter of 10 mm and a height of 10 mm, under displacement control with a crosshead speed of 0.5 mm/min. The elastic modulus of the Mg scaffold was calculated from the slope of the linear elastic stage of the stress-strain curve, and the yield strength was determined using the 0.2% offset method.

### 2.5 Degradation experiment

To evaluate the degradation behavior of the porous alloy, the magnesium alloy was immersed in Hank’s solution at 37°C for 5 days. The volume of gas produced was periodically recorded to assess the degree of hydrogen evolution. Additionally, the surface morphology post-degradation was observed macroscopically and microscopically using a stereomicroscope and SEM, respectively.

### 2.6 Preparation of zinc ion photoinitiator

Appropriate amounts of anhydrous zinc sulfate (ZnSO4) powder were dissolved in double-distilled water to prepare solutions with zinc ion (Zn^2+^) concentrations of 5*10^–3^ mol/L, 5*10^–4^ mol/L, and 5*10^–5^ mol/L 0.05 g of lithium phenyl-2,4,6-trimethylbenzoylphosphinate (LAP) was added to 10 mL of each solution, heated in a water bath at 40°C–50°C for 15 min with occasional shaking, to obtain photoinitiators with different zinc ion concentrations.

### 2.7 Preparation of Zn-AlgMA hydrogel

Alginate Methacryloyl (AlgMA) was purchased from Engineering For Life (EFL-AlgMA-50K). 10 mL of each zinc ion-concentrated photoinitiator was taken in 15 mL centrifuge tubes, wrapped in tin foil to avoid light, and then 0.5 g of AlgMA was added to each tube. The mixture was stirred at room temperature until the solution became clear and transparent, allowing Zn^2+^ in the initiator to form ionic bonds with the anions in AlgMA, resulting in ionic crosslinking. The ionically crosslinked AlgMA solution was sterilized using a 0.22 μm sterile syringe filter and stored at 4°C in the dark. When used, an appropriate amount of ionically crosslinked AlgMA solution was taken in a mold under aseptic conditions in a clean bench, and cured with a 405 nm light source for 10^–3^0 s to form the Zn-AlgMA hydrogel. The resulting hydrogel was placed at −80°C for 24 h, then freeze-dried in a vacuum freeze dryer for 3 days. The samples, after gold sputtering, were observed under a scanning electron microscope to examine their microstructure.

### 2.8 Ion Release Test (ICP)

Zn-AlgMA hydrogels with varying zinc ion concentrations were immersed in double-distilled water at a 1:10 volume ratio (as per the national standard GB/T 16886.12—XXXX/ISO 10993–12:2021) and incubated in a 37°C constant temperature shaker. On the first, fourth, seventh, and 10th days post-immersion, 1 mL of the immersion liquid was collected in new centrifuge tubes, diluted tenfold with double-distilled water, and the concentration of zinc ions in the immersion liquid was determined using an Inductively Coupled Plasma Mass Spectrometer (ICP-MS). All tests were conducted in triplicate to ensure repeatability and accuracy of results.

### 2.9 Water contact angle measurement

Zn-AlgMA hydrogels of appropriate size (length and width greater than 2 × 2mm) with different zinc ion concentrations were placed on a JY-82 contact angle measurement platform. The device’s automatic titration system was used to drop a corresponding reagent (such as a water droplet), and test photos were taken. The contact angle was measured using the tangent method/fitting method/goniometric method. In the captured images, a tangent line was drawn at the intersection point of the sample surface and droplet, intersecting with the sample’s horizontal plane, and the angle formed was automatically calculated by the computer, representing the contact angle.

### 2.10 Compression modulus measurement

The compression modulus of Zn-AlgMA hydrogels was determined using an electronic universal testing machine (CMT4204, Shenzhen Sansi Zongheng Technology Co., Ltd). Uniaxial compression tests were performed to determine the compressive strength and compression modulus of different hydrogel samples, using molds to prepare cylindrical hydrogel samples with a diameter of 20 mm and thickness of 10 mm. A 500 N sensor was used, and the samples were subjected to uniaxial compression at a strain rate of 10 mm/min until complete fracture. The compressive strength and compression modulus were measured, with each set of experiments repeated three times.

### 2.11 Rheological testing

The rheological properties of Zn-AlgMA hydrogels were tested using a HAAKE rheometer at 37°C and a gap of 1 mm between plates. A frequency of 1 Hz and a stress (τ) of 10 Pa were set, and tests were conducted in an oscillatory time sweep mode. With time as the horizontal axis and the storage modulus (G′) and loss modulus (G″) as the vertical axis, the changes in G′ and G″ over time t were monitored, with the gel time corresponding to the time when G′ equals G''. In stress sweep mode, stress scans were conducted at a constant frequency (1 Hz), with stress (τ) as the horizontal axis and G′ and G″ as the vertical axis. The yield stress value of the hydrogel was determined through the intersection of G′ and G''. In frequency sweep mode, tests were performed at a constant stress (τ = 10 pa) within an angular frequency (ω) range of 1–100 rad s^-1^. With ω as the horizontal axis and G′ and G″ as the vertical axis, the changes in G′ and G″ with ω were monitored, allowing for calculation of the complex shear modulus (|G*|) and complex viscosity (|η*|) at specific angular frequencies.

### 2.12 Preparation of biomimetic honeycomb-like scaffold

A small amount of Zn-AlgMA hydrogel, not exposed to UV light, was placed in a transparent mold, irradiated with UV light through the bottom of the mold for 3–5 s, then a DCPD-coated magnesium alloy was placed flat in the center of the mold, above the hydrogel. An appropriate amount of Zn-AlgMA hydrogel, also not exposed to UV light, was added to fill the mold. The mold was gently shaken to ensure the magnesium alloy was completely filled with hydrogel, then irradiated with UV light for 30–60 s for complete solidification, yielding the honeycomb-like scaffold. The obtained scaffold was immersed in simulated body fluid to assess its corrosion rate through hydrogen evolution experiments and magnesium ion release rate was detected through Ion Release Test (ICP).

### 2.13 Preparation of immersion liquid

Following the national standard GB/T 16886.12—XXXX/ISO 10993-12:2021, DCPD magnesium alloy, Zn-AlgMA hydrogel, or Zn-AlgMA@Mg scaffold were ultrasonically cleaned in deionized water, placed in closed containers, and immersed in conventional sterile DMEM complete culture medium at a 1:10 (w/v or v/v) ratio. The containers were incubated in a 37°C incubator for 24 h. After incubation, the liquid portion was collected and filtered through a 0.22 μm filter to obtain sterile immersion liquid.

### 2.14 Biocompatibility testing

#### 2.14.1 Cell proliferation experiment

BMSCs cultured in material immersion liquid were density adjusted to 1 × 104 cells/ml, uniformly seeded into 96-well plates, with 100 μL added to each well. Cells were incubated at 37°C and 5% CO2 for 24 h. After 24 h, 10ul of CCK-8 solution (KGA317, KeyGEN BioTECH, China) was directly added to each cell-containing well and incubated for an additional 2–4 h under the same conditions. The duration depended on cell type and proliferation speed. Post-incubation, absorbance was measured at 450 nm using a microplate reader. To ensure accuracy, wells containing medium and CCK-8 without cells were used as a blank control to calculate cell viability and proliferation rate.

#### 2.14.2 Cytotoxicity experiment

BMSCs were cultured in material immersion liquid. Once cells reached 70%–80% confluence in 96-well plates, the medium was removed and cells were washed with PBS. Then, a staining solution comprising Calcein-AM and propidium iodide (PI) (KGAF001, KeyGEN BioTECH, China) was added to each well and incubated for 30 min at 37°C. Calcein-AM is converted into green fluorescence by live cells, while PI stains dead cell nuclei red. Post-incubation, cells were observed and images captured using a fluorescence microscope to analyze the live/dead status of cells on the alloy scaffold. The magnitude of cytotoxicity was quantified by analyzing the fluorescence intensity of live *versus* dead cells using ImageJ software.

### 2.15 Antibacterial property testing

#### 2.15.1 Inhibition testing

Scaffolds for respective groups (Negative Control, Positive Control, 10^–3^ mol/L Zn-AlgMA, 10^–4^ mol/L Zn-AlgMA, 10^–5^ mol/L Zn-AlgMA, DCPD coated Mg alloy) were prepared as needed. Colonies of *Staphylococcus aureus*/*E. coli* were cultured in liquid medium, adjusted to the required concentration (0.5 McFarland standard), and co-cultured with the tested materials in centrifuge tubes for 24 h. After 24 h, the bacterial liquid was inoculated onto solid medium, incubated at 37°C for 24 h, and photographed for observation.

#### 2.15.2 Inhibition zone testing

Scaffolds for respective groups were prepared as needed and immersed in sterile saline for 24 h according to the national standard GB/T 16886.12—XXXX/ISO 10993–12:2021 to obtain respective immersion liquids. Filter paper discs (6 mm diameter) were soaked in each group’s immersion liquid for 24 h. A small amount of *S. aureus*/*E. coli* was transferred from the slant tube to liquid medium and incubated on a shaker. After a certain period, a specific amount of bacterial suspension was mixed with agar medium cooled to 40°C–45°C, poured onto plates, and solidified. The soaked filter paper discs were then placed on the plates, with one drop of material immersion liquid directly added on top, and incubated for 24 h to observe the size of the inhibition zone.

### 2.16 *In Vitro* repair effect

#### 2.16.1 Alizarin Red staining

BMSCs were cultured in osteogenic immersion liquid until appropriate growth density was achieved, then the medium was removed and cells were washed three times with PBS. Cells were fixed with 4% formaldehyde for 30 min, followed by another PBS wash. Cells were then exposed to 0.1% Alizarin Red staining solution (KGA363, KeyGEN BioTECH, China) for 30 min to ensure thorough staining. Excess stain was removed with PBS washes for clear background. Stained cells were observed under an optical microscope, with Alizarin Red binding to sulfated proteoglycans displaying red color. Three fields were randomly selected under ×4 microscope magnification, and the number of calcium nodules in the fields was counted and the mean value was used for quantitative contrast.

#### 2.16.2 Alkaline phosphatase staining

BMSCs cultured in osteogenic immersion liquid were washed thrice with PBS after reaching suitable density. Cells were fixed in 4% formaldehyde for 30 min, followed by a PBS wash to remove excess formaldehyde. Cells were then placed in alkaline phosphatase staining solution (KGA353, KeyGEN BioTECH, China), maintained for the required time per manufacturer’s instructions for thorough staining. Excess staining solution was removed by PBS wash, and stained cells were observed under an optical microscope. Three fields were randomly selected at ×4 microscope magnification, and the amount of cobalt sulfide staining in the fields was counted and the mean value was used for quantitative contrast.

#### 2.16.3 Alcian Blue staining

BMSCs cultured in chondrogenic immersion liquid were washed thrice with PBS after reaching the desired growth density. Cells were fixed in 4% formaldehyde for 30 min, followed by another PBS wash. Cells were then exposed to 1% Alcian Blue staining solution for 40 min to ensure complete staining. Excess stain was removed with PBS washes. Under the optical microscope, Alcian Blue binding to collagen displayed blue color. Three fields were randomly selected at ×4 microscope magnification, and the number of blue patches in the field was counted and the mean value was used for quantitative contrast.

#### 2.16.4 RT-PCR

BMSCs cultured in osteogenic/chondrogenic immersion liquid were collected and purified for total RNA, ensuring purity and quality. Using a one-step RT-PCR kit (11753100, Thermo Fisher, USA), RNA template, specific primers, and necessary reagents were mixed in a reaction tube. The reaction mixture underwent specific thermal cycles in a PCR instrument: initial reverse transcription at 50°C for 30 min for cDNA synthesis, followed by a pre-denaturation at 95°C for 5 min to prepare for PCR amplification. The mixture then underwent 40 cycles, each including denaturation at 95°C for 15 s, then annealing and extension at 60°C for 1 min, ensuring effective amplification of the target gene. Post-reaction, PCR products were quantitatively analyzed for target gene expression levels using real-time PCR fluorescence detection.

#### 2.16.5 Western blot

BMSCs cultured in osteogenic/chondrogenic immersion liquid to the desired growth density were washed 2–3 times with PBS, and cell lysis buffer pre-mixed with protease inhibitors was added. Cells were lysed for 20 min in an ice bath, using a cell scraper to ensure homogenization. The homogenate was then transferred to centrifuge tubes, vortexed for 30 min in an ice bath with repeated pipetting for thorough lysis. After centrifugation at 4°C and 12000 rpm for 15 min, the supernatant was collected. The supernatant was mixed with 5x loading buffer and heated at 98°C for 10 min. After preparing 10% separation gel and 5% stacking gel, the separation gel was poured first and bubbles removed, solidified, then the stacking gel added. After electrophoresis, the PVDF membrane was activated in methanol for 1 min, then the electrophoresed proteins were transferred to this membrane, avoiding bubble formation, and transferred in an ice bath for 1 h. The transferred PVDF membrane was blocked in 5% skim milk for 1 h, then incubated overnight at 4°C with diluted primary antibody, followed by washing with TBST and incubation with diluted secondary antibody for 1 h. After further washing, newly prepared luminous liquid was added for luminescent detection to observe protein expression.

It is worth noting that all the transferred PVDF membrane was trimmed into different bands according to the molecular weight of the target protein before incubated with primary antibody, which ensured that each target protein reacted only with the responding antibody without a cluttered background. In this case, clean and clear results can be obtained after exposure for semi-quantitative analysis; however, the full blots cannot be supplied for this article.

### 2.17 Proteomic analysis

BMSCs were cultured in osteogenic and chondrogenic induction culture medium immersion liquids containing biomimetic honeycomb-like scaffolds. After 14 days, protein was extracted from three sample groups (each group n = 3) and analyzed using Reverse-Phase High-Performance Liquid Chromatography (RP-HPLC). The proteomic data were qualitatively and quantitatively processed in MaxQuant/Andromeda software (version 1.3.0.5). Data analysis was conducted on platforms such as DAVID, String, Cytoscape, and OmicStudio (https://www.omicstudio.cn/tool). Differential protein expression was defined as *p* < 0.05. All proteomic data have been uploaded to the iProX database (http://www.iprox.cn/, Protein ID: IPX0007755000).

### 2.18 *In Vivo* repair effect

#### 2.18.1 Animal modeling

Following ethical review and approval by the First Hospital of Nanjing, this experimental protocol was authorized (DWSY-22143269). Ten New Zealand white rabbits were selected for this experiment. Preparation of Zn-AlgMA, magnesium metal, and Zn-AlgMA@Mg scaffolds was conducted, ensuring strict aseptic conditions prior to surgery. After administering general anesthesia to the rabbits, layered dissection exposed the femoral condyle area. A hole (diameter 6mm, depth 2 mm) was drilled at this site using an electric drill. Materials were then implanted according to predetermined groupings (blank control, Zn-AlgMA, DCPD-Mg alloy, Zn-AlgMA@Mg scaffold), with 3-4 replicates per group, followed by layer-by-layer suturing. Postoperative antibiotic injections were administered intramuscularly for three consecutive days. After 10 weeks, all experimental animals were euthanized, and the modeled areas were harvested for subsequent analysis.

#### 2.18.2 Gross and histological analysis

Following specimen retrieval, gross observation was conducted using a stereomicroscope; the samples were then fixed in formaldehyde and decalcified with EDTA solution at 37°C for 1 month. Subsequently, the samples were embedded in paraffin and sectioned. To identify the type of newly formed tissue, three staining methods were employed: Hematoxylin and Eosin (H&E), Safranin O/Fast Green (S/O), and Masson’s trichrome staining.

### 2.19 Statistical analysis

In this experiment, all data were presented as mean ± standard deviation (SD, N ≥ 3). Statistical analysis was performed using GraphPad Prism nine software for Windows (version 9.4.1, GraphPad Software Inc., USA). Differences between groups were assessed using one-way Analysis of Variance (ANOVA) and Tukey’s multiple comparison test, while comparisons between two groups were evaluated using t-tests. Differences were considered statistically significant when *p* < 0.05.

## 3 Results and discussion

### 3.1 Preparation and characterization of biomimetic honeycomb-like structure

**FIGURE 1:**
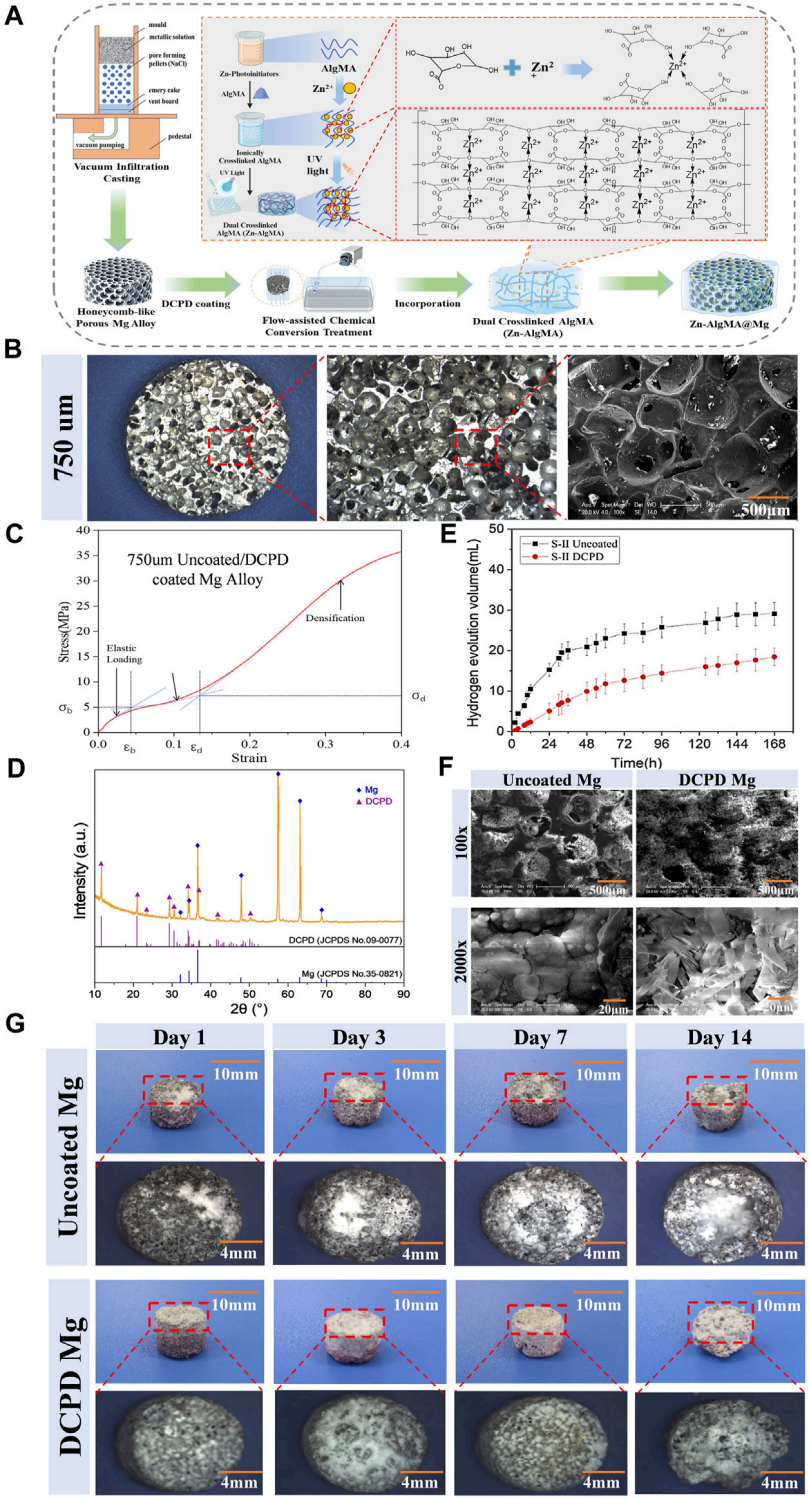
Fabrication and Characterization of the Biomimetic Scaffold **(A)** Schematic diagram representation of the biomimetic scaffold fabrication process: Magnesium-calcium alloy is combined with a porogen to create a porous structure followed by DCPD coating. Incorporation of Zn-AlgMA hydrogel is shown, resulting in the final honeycomb-like scaffold. **(B)** Macroscopic and SEM images of the porous architecture of the scaffold. **(C)** Stress-strain curve of the DCPD-coated magnesium alloy. **(D)** XRD patterns of uncoated Mg, DCPD-coated Mg. **(E)** Hydrogen evolution volume of uncoated and DCPD-coated Mg alloys over time. **(F)** Scanning Electron Microscope (SEM) images at ×100 and ×2000 magnifications show the surface morphology of uncoated and DCPD coated Mg scaffolds. **(G)** Sequential images over a 14-day period depicting the corrosion progression in uncoated Mg and DCPD-coated Mg scaffolds, with corresponding macroscopic changes and dimensional stability assessment.

In this experiment, a biomimetic honeycomb-like scaffold’s metallic structure was fabricated by uniformly mixing a molten magnesium-calcium alloy (99.8% Mg mixed with 0.2% Ca) with a porogen (NaCl particles of 750 μm size) and then processing it through a vacuum infiltration casting device to create a magnesium-calcium alloy with uniformly distributed 750 μm pores, followed by coating with a degradation-resistant DCPD layer using Flow-assisted chemical conversion treatment (Figure 1A).

The physicochemical properties of the biomimetic metallic scaffold (porous magnesium alloy) are crucial (Mohanasundaram et al., 2023). As shown in Figure 1B, the prepared magnesium alloy macroscopically appeared as 6 mm diameter cylindrical bodies. The micro-ct was scanned to measure the pore size of this scaffold (Supplementary Figure S3), and the results displayed that main pore size is 650–700 μm, the interconnected pore size is 100–200 μm, and the specific surface area is 35.32% ± 0.04%, and the porosity is 65.07% ± 0.51% (Supplementary Table S1). SEM scanning also supported these results that the surface and interior of the magnesium alloy possessed regularly distributed spherical pores with good interconnectivity, exhibiting typical open-pore structure characteristics. The porous magnesium alloy had a porosity of approximately 65.07%, with uniform pore sizes ranging from 600 to 700 μm. The interconnected pore size varied between 100 and 200 μm, and the specific surface area was measured at 35.32 mm^2^. Such pore sizes and porosity rates are similar to the porosity of human trabecular bone (40%–95%) (Morgan et al., 2018), providing an optimal microenvironment for cell migration and proliferation without compromising the scaffold’s mechanical strength (Muir et al., 2021). The stress-strain curve (Figure 1C) of the 750 μm magnesium alloy exhibited three typical sections: elastic region, yield plateau, and densification zone. Elastic modulus of human cancellous bone is about 0.02GPa–0.64 GPa and the compressive strength is 0.9–8.8 Mpa (Li et al., 2014; He et al., 2021). Thus, the mechanical properties of scaffolds are well-matched with those of the human cancellous bone.

Due to magnesium’s highly reactive chemical nature, it is prone to corrosion in both acidic and alkaline liquids (Zeng et al., 2018). Since bone regeneration typically requires at least 12 weeks (Ansari, 2019), most magnesium alloy implants would have degraded and lost their function within this period. Therefore, enhancing the corrosion resistance of magnesium alloys is a primary research focus. Common methods to improve the corrosion resistance of magnesium alloys include alloying, surface treatment, increasing purity, and composite materials (Tsakiris et al., 2021), among which surface treatment techniques hold unique advantages (Liu et al., 2021) and have become a hot research topic. Compared to other coatings, depositing a DCPD layer on magnesium alloy is easier, and it can transform into uniform HAP after 2 h immersion in NaOH solution (Dong et al., 2021). In this experiment, a DCPD coating was prepared on the surface of the 750 μm magnesium alloy using an electro-deposition method (Figure 1A). XRD diffraction analysis showed that the surface-treated magnesium alloy mainly consisted of an α-Mg matrix and DCPD secondary phase, with the three strongest diffraction angles of α-Mg at 50.703°, 50.107°, and 48.985°, and those of DCPD at 117.959°, 112.473°, and 104.497° (Figure 1D).

To validate the alteration in degradation rate of magnesium alloys post-DCPD coating, *in vitro* immersion degradation experiments were essential. In our experiment, uncoated magnesium alloy was used as a control, and the macroscopic appearance and hydrogen gas evolution of the magnesium alloys were analyzed after immersion for 1d, 3d, 7d, and 14d (Figures 1E, G). Upon immersion in Hank’s solution, hydrogen gas bubbles immediately appeared on the surface of the uncoated magnesium alloy, and with time, the surface accumulated many white corrosion products. In contrast, DCPD-coated magnesium scaffold exhibited lower hydrogen evolution volume than the uncoated one at the initial immersion stage, and a similar hydrogen evolution rate to the uncoated one with the increase of immersion time. Herein, the rapid degradation of magnesium scaffolds might cause hemolysis owing to local high alkalinity. Influence of Mg^2+^ concentration, pH value and specimen parameter on the hemolytic property of biodegradable magnesium, and it is meaningful to inhibit the corrosion rate at the initial immersion stage by DCPD coating. Furthermore, the uncoated magnesium alloy exhibited flaking and defects after 3 days of immersion, with decomposition becoming more pronounced over time, while the DCPD-coated alloy samples began to show flaking only after 14 days, indicating the DCPD coating’s superior ability to slow down the degradation of magnesium alloy (Rahman et al., 2020).

Further investigation was conducted on the surface morphology of the DCPD-coated magnesium alloy (Figure 1F). SEM analysis revealed that at ×100 magnification, the uncoated magnesium alloy surface had a typical porous structure with numerous uniformly distributed, interconnected spherical pores connected by smaller pores ranging from 100–200 μm, forming a complete open-cell foam structure. Post-DCPD coating, the spherical pores on the magnesium alloy surface were partially obscured, with a corresponding reduction in the number of small pores responsible for interconnectivity. At ×2000 magnification, the uncoated magnesium alloy surface exhibited coarse and loose particles; meanwhile, the DCPD-coated magnesium alloy displayed a smooth, flat, and dense surface. The coating surface had cracks and gaps, but its smooth surface consistently facilitated cell adhesion.

In summary, pore size, shape, and distribution have complex impacts on the structure and properties of porous materials (Su et al., 2023). Pore size and porosity not only determine the microenvironment for cell migration and proliferation but also play a decisive role in the scaffold’s mechanical properties. The honeycomb-like magnesium alloy pores in this experiment were within the range suitable for cell growth (Chen et al., 2020), with its porous structure maintaining an elasticity modulus similar to human bone (Wang et al., 2021) and facilitating metabolic exchange to improve the microenvironment (Zhang et al., 2023). Mg, being one of the most electrochemically active metals with a very low standard electrode potential, easily degrades in Cl-containing solutions (such as bodily fluids) to form magnesium ions (Mei et al., 2019), which are absorbed by surrounding tissues and excreted via urine if in excess (Lin et al., 2002). A controlled degradation rate of magnesium alloy materials does not adversely affect the human body, while rapid degradation can lead to rapid hydrogen gas release, causing gas cavitation (Kopp et al., 2023). Therefore, this experiment incorporated a DCPD coating on the magnesium alloy surface to reduce its degradation rate, consistent with published results (Gao et al., 2021). The successful application of the DCPD coating on the magnesium alloy provided an effective protective layer, thus slowing its degradation in a biological environment. When the magnesium alloy came into contact with the biological environment, the coating acted as a barrier, limiting the direct reaction of magnesium with environmental substances (such as water and oxygen) (Zhang et al., 2021), thereby reducing hydrogen gas production and formation of corrosion products. This explains why the DCPD-coated magnesium alloy initially produced almost no gas bubbles upon immersion. Additionally, the DCPD coating can transform into HAP in alkaline environments (Braga et al., 2022). As a major component of bone tissue, HAP’s stability and biocompatibility are relatively high, further enhancing the coating’s anti-degradation effect.

### 3.2 Preparation and characterization of biomimetic “honey” matrix

In the repair of osteochondral defects, the process of cartilage repair is distinctly different from that of bone repair. Cartilage tissue primarily consists of a small fraction of cells and other major components such as type II collagen and proteoglycans (Vonk et al., 2010), which harder metallic scaffolds cannot effectively mimic as an extracellular matrix of cartilage tissue (Yi et al., 2022). Inspired by nature’s honeycomb-like filled with “honey,” overlaying a metallic scaffold with an extracellular matrix conducive to cartilage tissue growth can effectively address this challenge. Sodium alginate, a natural polysaccharide carbohydrate, is widely regarded as a promising biomaterial in articular cartilage tissue engineering due to its structural similarity to cartilage tissue and its degradability via hydrolysis and enzymatic pathways in the body (Abka-khajouei et al., 2022). Considering the non-invasive, spatiotemporally controllable, safe, and excellent physical properties of photo-responsiveness (Li et al., 2019), along with the richness of zinc ions in honey (Ajibola et al., 2012) and the properties of Zn^2+^ in promoting cartilage regeneration (Ciaffaglione and Rizzarelli, 2023), this experiment designed a zinc ion dual-crosslinked sodium alginate hydrogel (Zn-AlgMA). Solutions of different concentrations of ZnSO4 were mixed with LAP to form various photoinitiators, and these Zn^2+^ containing photoinitiators were mixed with AlgMA monomer. The divalent cations (Zn^2+^) in the photoinitiator formed ionic bonds with the anions in AlgMA molecules, fixing the biomacromolecules together to form a primary crosslinked structure. Subsequently, the Zn-AlgMA hydrogel was irradiated under UV light for 30 s, where the photoinitiator in the solution absorbed light energy to generate free radicals, thereby bonding AlgMA molecules to form a solid gel, serving as the cartilage repair part of the biomimetic honeycomb-like scaffold (simulating “honey”) (Figure 1A).

**FIGURE 2:**
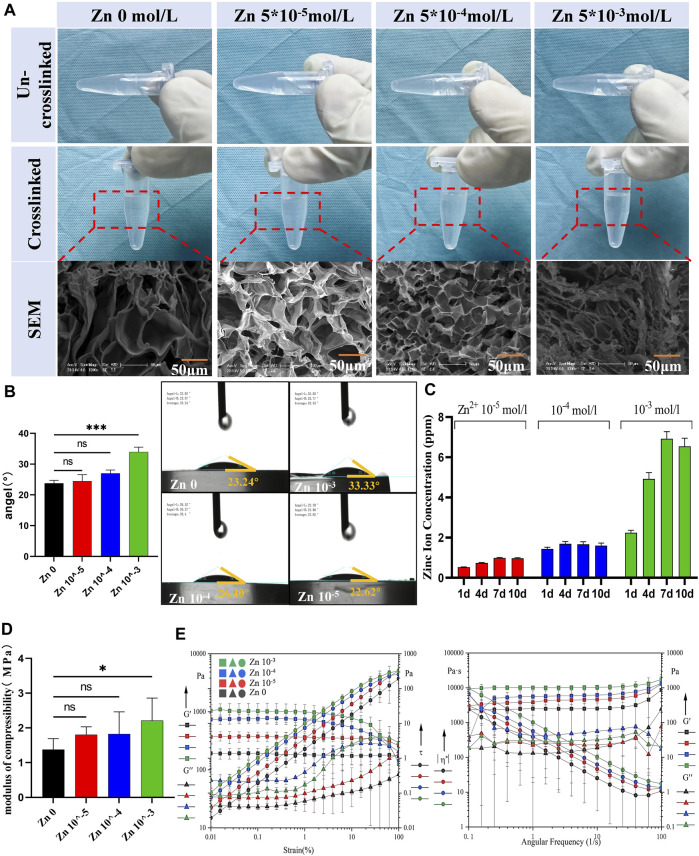
Preparation and characterization of Zn-AlgMA hydrogels **(A)** Visual and SEM comparisons of the Zn-AlgMA hydrogels at different zinc ion concentrations. **(B)** Water contact angle measurements demonstrating the hydrophilicity of the Zn-AlgMA hydrogels with various concentrations of Zn^2+^. **(C)** The release kinetics of zinc ions from the hydrogels over time. **(D)** Compressive modulus data for Zn-AlgMA hydrogels. **(E)** Rheological properties of the Zn-AlgMA hydrogels, with graphs representing the storage modulus (G′) and loss modulus (G″) across strain and angular frequency sweeps.

Three different concentrations of zinc ion Zn-AlgMA were prepared and characterized in this experiment. For ease of description, AlgMA hydrogels with Zn^2+^ concentrations of 0 mol/L, 5*10^–3^ mol/L, 5*10^–4^mol/L, and 5*10^–5^ mol/L are hereafter referred to as Zn 0, Zn 10^–3^, Zn 10^–4^, and Zn 10^–5^, respectively. Macroscopically, the solely ionically crosslinked Zn-AlgMA hydrogel appeared as a transparent, viscous liquid at room temperature, with varying Zn^2+^ concentrations having minimal impact on its macroscopic morphology (Figure 2A). No significant differences in the macroscopic appearance of different concentrations of Zn-AlgMA were observed post-solidification. SEM analysis showed larger pores in the Zn 0 group hydrogel, which gradually diminished and densified as zinc ion concentration increased (Figure 2A). As more Zn^2+^ is added to AlgMA, it resulted in more second cross-linking points and increase the cross-linking density. This variation in pore structure is vital for cell migration and growth, where smaller pores may limit internal cell movement but simultaneously offer a higher surface area conducive to cell adhesion and proliferation (Murphy et al., 2010).

To ascertain the suitability of Zn-AlgMA hydrogel as a scaffold for cartilage regeneration, this experiment evaluated its physical properties. The water contact angles experiment revealed (Figure 2B) that as the Zn^2+^ concentration increased, the water contact angles increased and changed to hydrophobicity gradually. An explanation to this is that with the Zn^2+^ concentration increased, its ionic cross-linked structure becomes tighter and the pores become smaller, leading to the water molecules decreased in the matrix, thus affecting the hydrophilicity of the hydrogel. ICP results showed (Figure 2C) that the Zn 10^–4^ and Zn 10^–5^ groups released Zn^2+^ at a relatively slow rate (<2 ppm) with minor variation in release rate over time. In contrast, the Zn 10^–3^ group exhibited the fastest release rate, potentially leading to excessively high local concentrations of zinc ions. Compression tests indicated (Figure 2D) that the addition of Zn^2+^ enhanced the compression resistance of AlgMA. Although there was no statistically significant difference between Zn 10^–3^, Zn 10^–4^, and the control group (Zn 0), the mechanical stability of AlgMA indeed improved with the addition of Zn^2+^, which is crucial for supporting the structural integrity of cartilage tissue. Additionally, rheological property testing revealed that as the Zn^2+^ concentration increased, both the storage modulus (G′) and loss modulus (G″) also increased, with minimal change in the linear region. This suggests that all four hydrogels could maintain a solid state well. Frequency sweep tests confirmed this, as the slopes of G′ and G″ against frequency were zero in the graph, indicating that all four hydrogels had weak frequency dependency and their internal structures were well-formed, effectively maintaining gel state (Figure 2E).

The results of the above experiments indicate that the Zn-AlgMA hydrogel prepared in this experiment exhibited favorable physicochemical properties, including a tight pore structure, altered hydrophilicity, slow release rate, and stable mechanical performance. These findings not only confirm the suitability of Zn-AlgMA hydrogel as a filling matrix for the biomimetic honeycomb-like scaffold but also highlight its potential application in effectively simulating the extracellular matrix of cartilage tissue in cartilage tissue engineering, thereby promoting cartilage repair and regeneration.

### 3.3 Biocompatibility and antibacterial properties of biomimetic scaffold components

**FIGURE 3:**
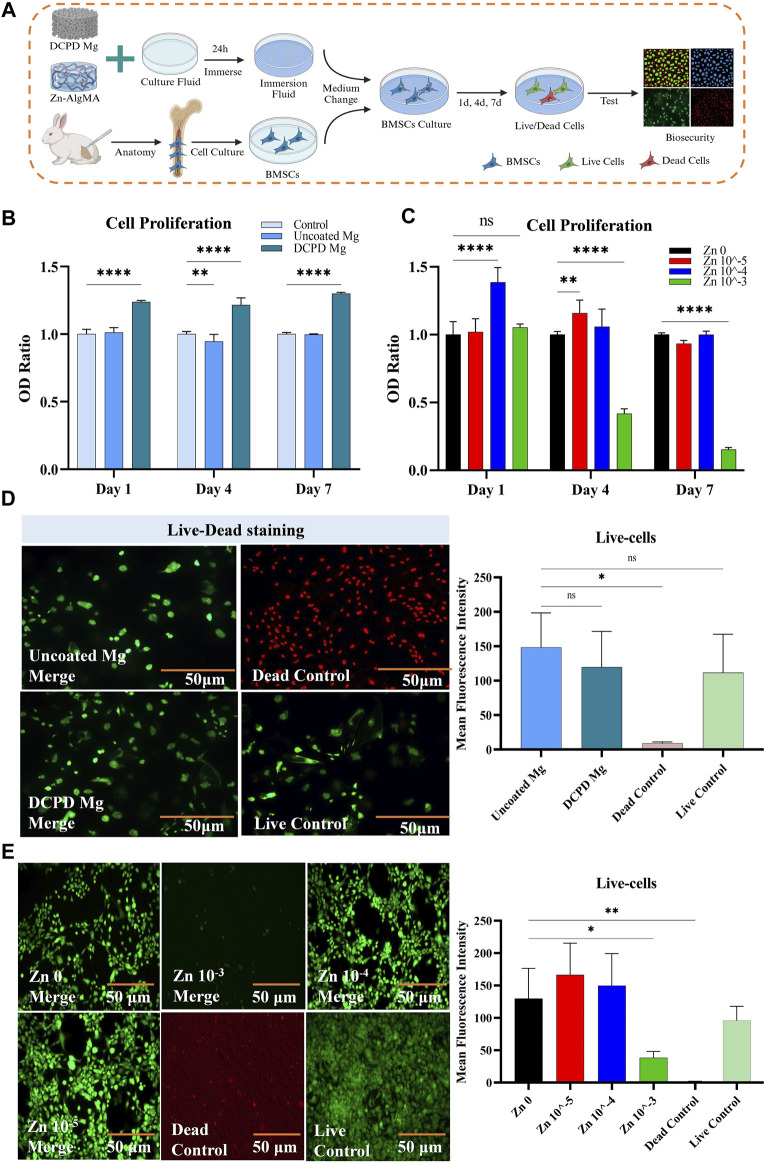
Biocompatibility of DCPD Mg and Zn-AlgMA **(A)** Schematic diagram of the biocompatibility test between DCPD Mg and Zn-AlgMA. **(B)** CCK-8 assay results demonstrating the viability and proliferative capacity of cells cultured with uncoated Mg and DCPD Mg scaffolds over a period of 7 days. **(C)** CCK-8 assay results for cells cultured with Zn-AlgMA hydrogels at varying concentrations of Zn^2+^. **(D)** Live-Dead staining images comparing the viability of cells in contact with uncoated Mg and DCPD Mg scaffolds. Live cells are stained green and dead cells are stained red. **(E)** Live-Dead staining images of cells cultured with Zn-AlgMA hydrogels at different Zn2+ concentrations. Live cells are stained green and dead cells are stained red. Note: *: *p* < 0.05; **: *p* ≤ 0.01; ***: *p* ≤ 0.001; ****: *p* ≤ 0.0001; ns: No significant.

As degradable tissue engineering biomaterials, the biocompatibility of each component of the scaffold is crucial. For clarity, uncoated magnesium alloy and DCPD-coated magnesium alloy were referred to as Uncoated Mg and DCPD Mg, respectively. This experiment conducted cell proliferation and cytotoxicity tests on Uncoated Mg and DCPD Mg as shown in the schematic diagram (Figure 3A). The cell proliferation assay (Figure 3B) revealed that Uncoated Mg did not exhibit significant proliferative effects over time, but following the application of the DCPD coating, its proliferation-enhancing effect was markedly increased. From a cytotoxicity perspective (Figure 3D), both Uncoated Mg and DCPD Mg showed no significant difference compared to the live cell control, demonstrating high biocompatibility.

Sodium alginate forms a gel with calcium and zinc ions, providing a controlled release effect (Hu et al., 2021). Although studies have found that Zn^2+^ can promote cartilage tissue regeneration, Zn^2+^ only maintains biocompatibility within a certain concentration range; excessively high local Zn^2+^ concentrations can exhibit significant cytotoxicity (Ng et al., 2017). In this experiment, cells were cultured with Zn-AlgMA hydrogel immersion liquid. Zn 10^–4^ showed obvious cell proliferation effect on the first day, and Zn 10^–5^ showed the best proliferation effect on day 4. Till day 7, there were no significant differences between Zn 10^–4^, Zn 10^–5^ and the control group. Zn 10^–3^ continued to show obvious growth inhibition all over the time (Figure 3C). Interestingly, cytotoxicity tests confirmed that Zn 10^–3^ was not conducive to cell growth, while Zn 10^–4^ and Zn 10^–5^ demonstrated good biocompatibility (Figure 3E).

**FIGURE 4:**
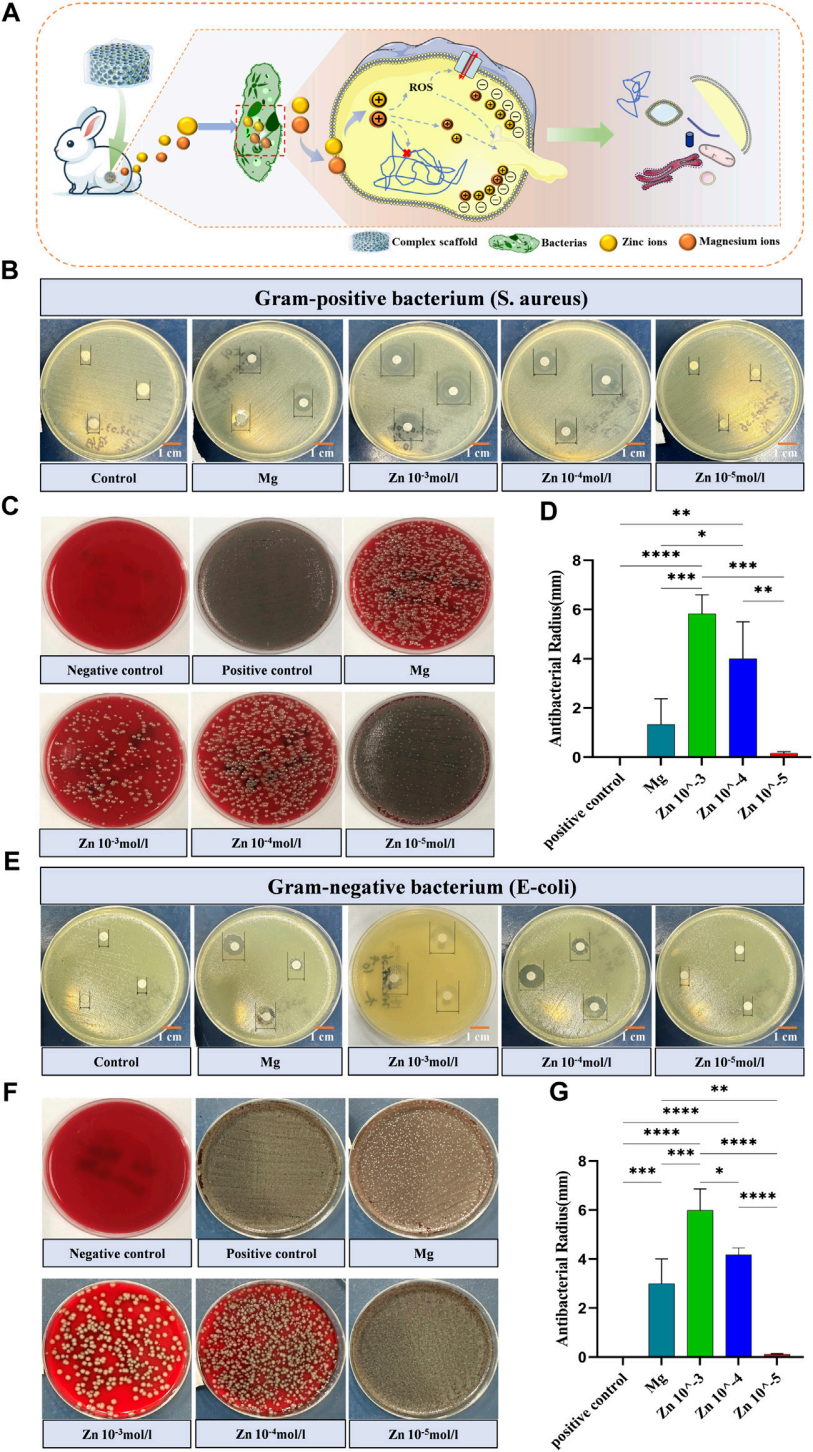
Antimicrobial activity of stent components **(A)** Schematic depicting the bactericidal mechanism by which the honeycomb-like scaffold releases embedded zinc and magnesium ions into the interior of bacteria. **(B)** Visual graph of the inhibition zone size of different scaffold components against Gram-positive bacteria (*Staphylococcus aureus*). **(C)** Colony-inhibitory effect of different scaffold components on Gram-positive bacteria (*Staphylococcus aureus*). **(D)** Quantitative comparison of the inhibition zone size of Gram-positive bacteria (*Staphylococcus aureus*) with different scaffold components. **(E)** Visual graph of the inhibition zone size of different scaffold components against Gram-negative bacteria (*Staphylococcus aureus*). **(F)** Colony-inhibitory effect of different scaffold components on Gram-negative bacteria (*Staphylococcus aureus*). **(G)** Quantitative comparison of the inhibition zone size of Gram-negative bacteria (*Staphylococcus aureus*) with different scaffold components. Note: *: *p* < 0.05; **: *p* ≤ 0.01; ***: *p* ≤ 0.001; ****: *p* ≤ 0.0001; ns: No significant.

In orthopedic surgery, infection is one of the most common and serious postoperative complications (Motififard et al., 2021), often leading to surgical failure and healing difficulties. This is particularly critical with joint cavity implants, where an infection can cause irreparable damage to the entire joint function (Kaufman et al., 2016). Honey in nature has been proven to have significant antibacterial effects (Albaridi, 2019), and zinc ions play an important role in its antibacterial properties, with the efficacy of these ions closely related to their concentration (Pasquet et al., 2014). In the honeycomb-like biomimetic scaffold constructed in this experiment, the DCPD magnesium alloy matrix and Zn-AlgMA hydrogel can respectively release magnesium and zinc ions, making it particularly important to evaluate the antibacterial properties of these two components. Antibacterial experiments demonstrated that the Zn 10^–3^ hydrogel exhibited strong antibacterial activity against both Gram-negative bacteria (*Escherichia coli*, *E. coli*) and Gram-positive bacteria (*Staphylococcus aureus*, *S. aureus*), while magnesium alloy showed certain antibacterial effects only against Gram-positive bacteria. The Zn 10^–4^ hydrogel had stronger antibacterial performance against both Gram-positive and Gram-negative bacteria compared to Zn 10^–5^ hydrogel, which almost had no effect (Figures 4C, F). Inhibition zone experiments revealed that the antibacterial efficacy of the hydrogels increased with the concentration of Zn^2+^, with magnesium alloy’s antibacterial performance falling between that of the Zn 10^–4^ and Zn 10^–5^ hydrogels (Figures 4B, D, E, G).

Antibacterial tests showed that the zinc ions released from the Zn-AlgMA hydrogel have significant antibacterial properties. Studies suggest that the antibacterial action of zinc ions is mainly achieved through three pathways (Figure 4A): First, zinc ions can inhibit bacterial ATP synthesis, disrupting the replication of genetic material (Godoy-Gallardo et al., 2021). Second, zinc ions can act as active catalytic centers, catalyzing the generation of hydroxyl radicals and reactive oxygen species from water or air molecules, triggering oxidative stress responses, damaging bacterial reproductive capabilities, and ultimately leading to their death (Mendes et al., 2022). Lastly, given that zinc ions are positively charged, when they encounter the negatively charged bacterial cell surface in excess, Coulomb forces cause the zinc ions to adhere firmly to the cell membrane. Subsequently, they penetrate the cell wall, causing cell wall rupture and cytoplasmic leakage, thereby disrupting bacterial reproduction and ultimately leading to bacterial death (Ye et al., 2020). Mg^2+^ exhibited antimicrobial effect because its rapid dissolution leaded to an increased PH value in the medium, resulting in the death of bacteria.

### 3.4 The role of honeycomb-like scaffold in promoting bidirectional differentiation of bone marrow mesenchymal stem cells

**FIGURE 5:**
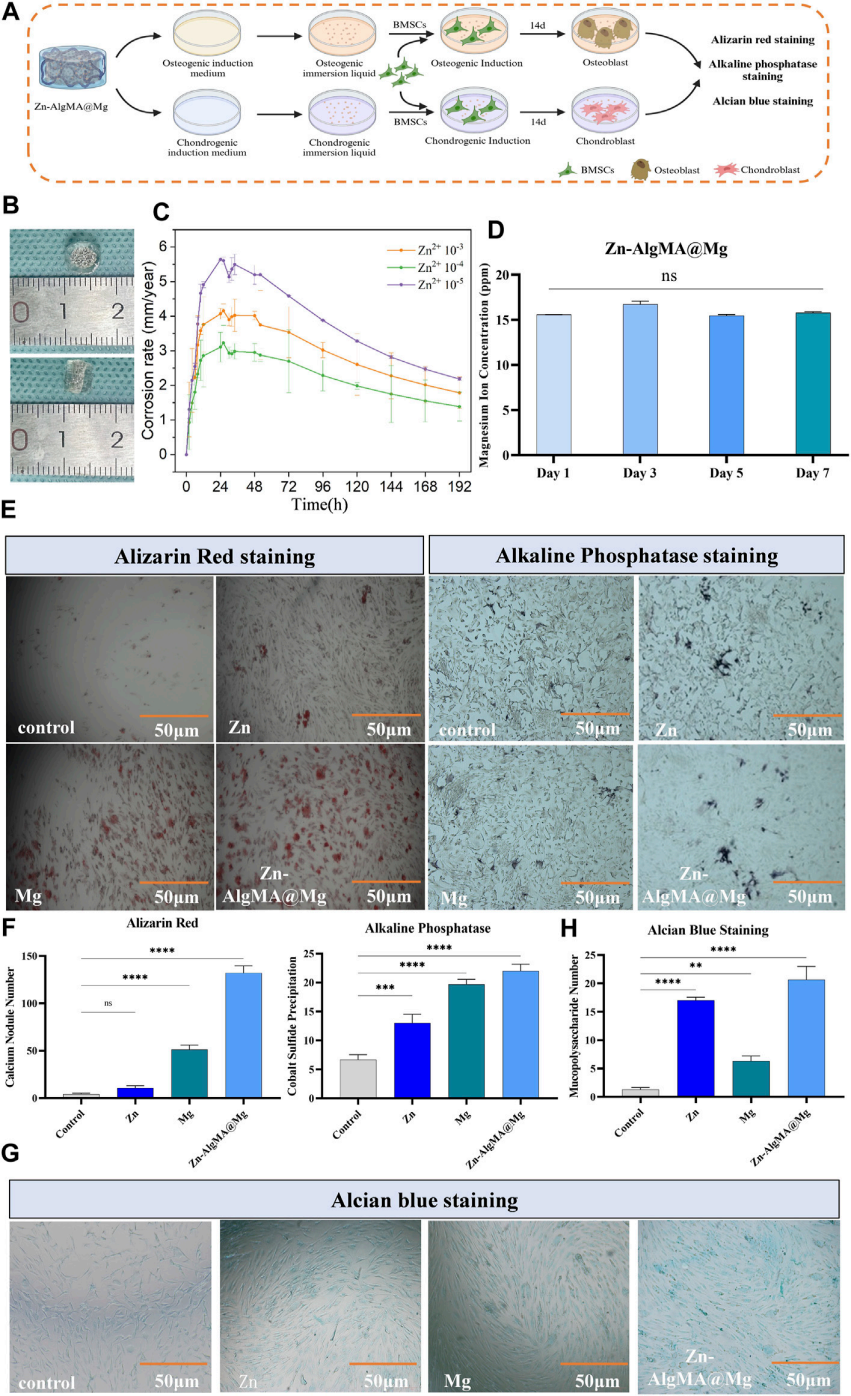
Degradation assessment of osteogenic and chondrogenic potential of honeycomb-like scaffold **(A)** Schematic diagram of the bidirectional differentiation performance test of the honeycomb-like scaffold *in vitro*. **(B)** Visual macroscopic comparison of honeycomb-like scaffolds with scaled references. **(C)** Corrosion rate over time for honeycomb-like scaffolds with different Zn2+ concentrations. **(D)** Concentration of magnesium ions released from honeycomb-like scaffolds over 7 days. **(E)** Histological staining of osteogenic differentiation after culturing honeycomb-like scaffold extracts under osteogenic induction culture conditions: Alizarin red staining shows calcium deposition, and alkaline phosphatase staining shows the early osteogenic activity of different scaffold treatments. **(F)** Alizarin red staining and ALP staining were quantitatively analyzed. **(G)** Alcian blue staining after culture of honeycomb-like scaffold extract in chondrogenic induction culture state. **(H)** Alcian blue staining was used for quantitative analysis. Note: *: *p* < 0.05; **: *p* ≤ 0.01; ***: *p* ≤ 0.001; ****: *p* ≤ 0.0001; ns: No significant.

Based on the aforementioned results, it is evident that DCPD-coated magnesium alloy, possessing superior mechanical properties and biocompatibility, is an ideal material for bone repair. Moreover, considering the biocompatibility, physical properties, and antibacterial performance of Zn-AlgMA hydrogel, it also provides an ideal microenvironment for cartilage regeneration. Consequently, this experiment encapsulated Zn-AlgMA hydrogel around the surface of DCPD-coated magnesium alloy to fabricate a honeycomb-like style biomimetic composite scaffold (abbreviation as Zn-AlgMA@Mg) (Figure 1A), and then bidirectional differentiation induction was performed to study its repair promotion performance *in vitro* (Figure 5A).

Macroscopically visible (Figure 5B), the biomimetic scaffold which prepared in the mold, is cylindrical, approximately 6 mm in diameter and 4 mm in height, with the hydrogel presenting a thinner layer at the bottom and a thicker layer at the top to provide an appropriate microenvironment for upper layer cartilage repair in osteochondral defects. The advantage of designing such a biomimetic scaffold in this way is that the upper thick hydrogel can simulate the cartilage matrix, while the magnesium alloy wrapped by the hydrogel can not only further reduce the degradation rate, but also provide mechanical support for bone repair and promote bone regeneration. The hydrogen evolution experiment (Figure 5C) found that the magnesium alloy degradation rate was fastest in the scaffold made with Zn 10^–5^ hydrogel, followed by Zn 10^–3^ hydrogel, while the magnesium alloy coated with Zn 10^–4^ hydrogel degraded the slowest. It is noteworthy that the degradation rate of magnesium alloy was further reduced when the alloy surface was covered with organic hydrogel (Figures 1E; Figure 5C), likely due to the organic polymer coating reducing contact between the magnesium alloy and water molecules, further slowing degradation (Wong et al., 2010). Based on these results, this experiment ultimately chose 10^–4^ mol/L Zn^2+^ hydrogel to form the honeycomb-like scaffold for further studies. For convenience, blank group, DCPD coated magnesium alloy, 5*10^–4^ mol/L Zn^2+^ AlgMA hydrogel and composite scaffold were abbreviated as Control, Mg, Zn and Zn-AlgMA@Mg respectively.

Since magnesium ions (Mg^2+^) released from magnesium alloy degradation are effective in promoting bone repair, this experiment examined the release rate of Mg^2+^ from the Zn-AlgMA@Mg scaffold, finding no significant difference as time progressed (Figure 5D). The ability of the Zn-AlgMA@Mg scaffold to promote differentiation of bone marrow mesenchymal stem cells (BMSCs) was then assessed. As calcium nodules and ALP are important early indicators of osteogenesis (Trivedi et al., 2020), after 2 weeks of culture in induced culture medium immersion liquid, it was found that the Zn-AlgMA@Mg scaffold had the strongest capability to promote osteogenesis, followed by magnesium alloy, with Zn-AlgMA’s osteogenic ability only slightly better than the control group (Figures 5E, F). Additionally, as cartilage repair is a crucial aspect of integrated osteochondral repair, the ability of the Zn-AlgMA@Mg scaffold to promote BMSC chondrogenic differentiation was also evaluated. Alcian Blue staining (Figures 5G, H) showed that the Zn-AlgMA@Mg scaffold had the highest expression of Collagen-II, followed by the Zn and Mg groups, both of which were superior to the control group.

These results indicate that the honeycomb-like style biomimetic scaffold constructed in this experiment demonstrates high potential for application in the field of osteochondral defects repair. The good mechanical properties and biocompatibility of the magnesium alloy, combined with the excellent biocompatibility, physical properties, and antibacterial performance of Zn-AlgMA hydrogel, form the foundation of this honeycomb-like scaffold’s biological performance.

### 3.5 Proteomic analysis of honeycomb-like Scaffold’s promotion of bidirectional differentiation of bone marrow mesenchymal stem cells

**FIGURE 6:**
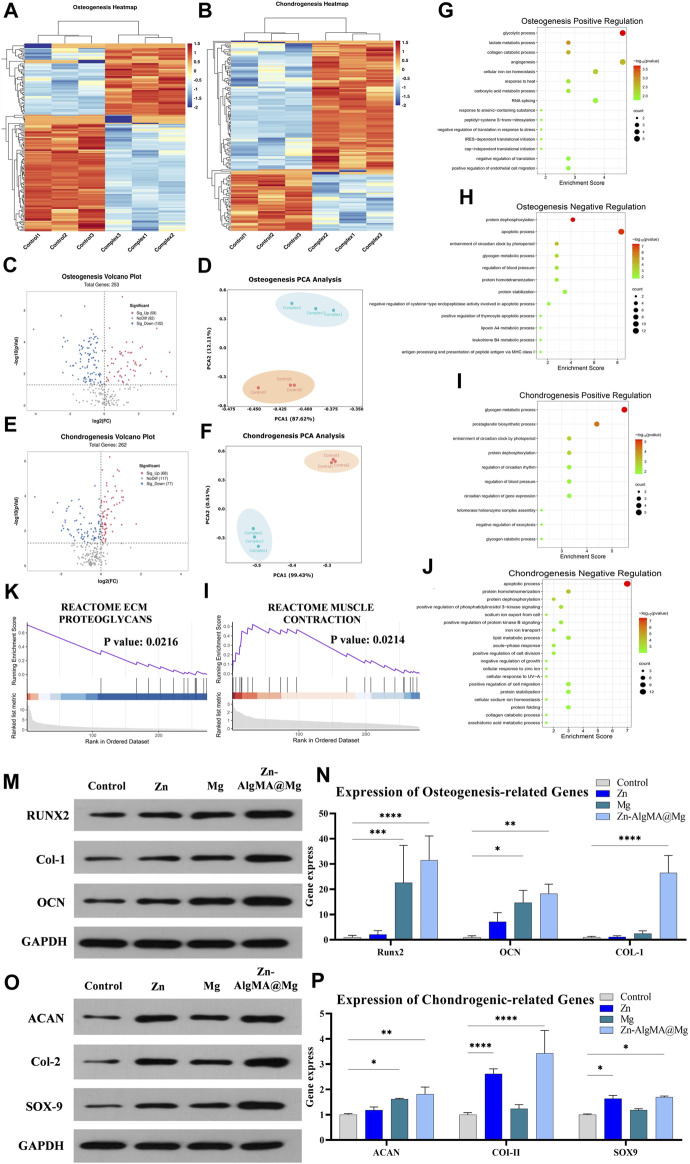
Analysis of the mechanism of Zn-AlgMA@Mg scaffold promoting bidirectional differentiation of osteogenesis and chondrogenesis **(A)** Heat map of gene expression related to osteogenesis. **(B)** Heat map of gene expression related to chondrogenesis. Volcano plot **(C)**, PCA plot **(D)**, GO enrichment analysis of upregulated genes **(G)**, GO enrichment analysis of downregulated genes **(H)**, and partial GSEA analysis **(K)** during osteogenic differentiation. Volcano plot **(E)**, PCA plot **(F)**, GO enrichment analysis of upregulated genes **(I)**, GO enrichment analysis of downregulated genes **(J)**, partial GSEA analysis **(L)** during chondrogenic differentiation. **(M)** Expression of osteogenesis -related proteins. **(N)** Osteogenesis-related gene expression. **(O)** Expression of chondrogenesis-related proteins. **(P)** Expression of chondrogenesis-related genes. Note: *: *p* < 0.05; **: *p* ≤ 0.01; ***: *p* ≤ 0.001; ****: *p* ≤ 0.0001; ns: No significant.

Proteomics was employed to analyze the potential mechanisms by which the honeycomb-like scaffold (Zn-AlgMA@Mg) influences the bidirectional differentiation of BMSCs. In this experiment, we compared the effects of Zn-AlgMA@Mg scaffold immersion liquid on BMSC differentiation in osteogenic and chondrogenic induction environments. Heatmaps illustrated differential protein expression (Figures 6A, B). PCA analysis revealed that the Zn-AlgMA@Mg scaffold had distinct effects on BMSC osteogenic and chondrogenic differentiation (Figures 6D, F). In osteogenesis, a total of 2,327 proteins were identified, with volcano plots displaying 161 differentially expressed proteins (Figure 6C); 59 were upregulated and 102 downregulated, marked in red and blue respectively. In chondrogenesis, 2,588 proteins were identified with 145 differentially expressed proteins, including 68 upregulated and 77 downregulated proteins (Figure 6E).

GO enrichment analysis further assessed the pathways impacted by the Zn-AlgMA@Mg scaffold. In osteogenesis, upregulated proteins were enriched in pathways closely related to osteogenic processes such as “glycolysis,” “angiogenesis,” “intracellular iron ion homeostasis,” “collagen catabolic metabolism,” and “stress regulation” (Figure 6G) (Balogh et al., 2018; Diomede et al., 2020; Claeys et al., 2021; Lin et al., 2022; Turishcheva et al., 2022). Western blot and RT-PCR results corroborated these findings, with Runt-related transcription factor 2 (RUNX2), Collagen-1 (Col-1), and Osteocalcin (OCN), as three key osteogenic markers in the aforementioned pathways, showing the highest gene and protein expression levels in the Zn-AlgMA@Mg group. However, trends varied among the three markers (Figures 6M, N): For RUNX2, the Zn group was only slightly higher than control, much lower than the Mg and Zn-AlgMA@Mg groups; for Col-1, both Zn and Mg groups were slightly higher than control, but combined Zn^2+^ and Mg^2+^ expression far exceeded the control; OCN expression incrementally increased in order. Downregulated osteogenic proteins were enriched in “protein dephosphorylation,” “cell apoptosis,” “carbohydrate metabolism” (Figure 6H) (Giner et al., 2013; Donat et al., 2021; Yang et al., 2022), consistent with previous CCK-8 and Live/dead staining (Figures 3B, D), suggesting the Zn-AlgMA@Mg scaffold promotes cell proliferation and maintains cell viability. In chondrogenesis, upregulated proteins were enriched in “glycogen metabolism,” “protein dephosphorylation,” “circadian rhythm regulation,” “prostaglandin synthesis” (Figure 6I) (Matta and Mobasheri, 2014; Caron et al., 2016; Hollander and Zeng, 2019; Alagha et al., 2021). Western blot and RT-PCR were used to validate key chondrogenic markers: Aggrecan (ACAN), Collagen-II (Col-II), SRY-box transcription factor 9 (SOX9). Results showed consistent trends in gene and protein expressions (Figures 6O, P): For Col-II, the Zn group’s expression was second only to the Zn-AlgMA@Mg group but higher than the Mg group, which was only slightly above control; ACAN expression incrementally increased across all groups; Zn-AlgMA@Mg group’s SOX9 expression was slightly higher than Zn, with Mg above control, aligning with Alcian Blue staining results (Figures 5G, H). Downregulated chondrogenic proteins were concentrated in “cell apoptosis,” “PI3K signaling pathway,” “collagen degradation,” “lipid metabolism” (Figure 6J) (Goldring, 2012; Honvo et al., 2020; van Gastel et al., 2020; Klampfleuthner et al., 2022), indicating the Zn-AlgMA@Mg scaffold might promote post-injury cartilage regeneration by maintaining microenvironmental homeostasis. Thus, the Zn-AlgMA@Mg scaffold comprising Zn-AlgMA and DCPD-Mg alloy demonstrates efficacy in promoting osteogenesis and chondrogenesis.

GSEA further elucidated molecular functions identified in GO enrichment analysis. In osteogenesis, upregulated proteins were enriched in “extracellular matrix proteoglycans,” “estrogen response,” and “endoplasmic reticulum signal transduction” (Figure 6K; Supplementary Figure S1A); in chondrogenesis, they participated in “muscle contraction,” “mitochondrial metabolism,” “estrogen response” (Figure 6L; Supplementary Figure S2A). Subsequently, differentially expressed proteins were used to construct Protein-Protein Interaction (PPI) networks; in osteogenesis, FKBP4, DCN, SLC9A1 emerged as important upregulated proteins (S1B). FKBP4, a member of the immunophilin protein family, plays a role in immunoregulation and fundamental cellular processes involving protein folding and transport. Previous studies have established a close association between the FKBP family and senile osteoporosis (Krakow et al., 2014). DCN, a gene encoding a protein, regulates collagen fibers, maintaining normal bone structure and strength in osteogenesis (Adachi et al., 2022). SLC9A1, a membrane transporter protein found in various tissues, primarily regulates sodium and hydrogen ion transmembrane transport and is closely associated with BMSC cell migration and volume maintenance (Iwama et al., 2018). In chondrogenesis, GBR1, AGL, etc., were key upregulated proteins in the PPI network (S2B). GBR1, involved in fatty acid metabolism as a GAPDH-dependent oxidoreductase (Morgan et al., 2017); AGL gene encodes amylo-1,6-glucosidase, 4-α-glucanotransferase (AGL), an enzyme crucial in skeletal muscle glycogen metabolism (Qu et al., 2020).

Overall, proteomic analysis confirmed that the Zn-AlgMA@Mg scaffold enhances the expression of proteins related to pathways involved in osteogenic and chondrogenic differentiation, and impacts apoptosis signaling, metabolic pathways, and extracellular matrix formation, promoting cell proliferation and differentiation.

### 3.6 *In Vivo* evaluation of honeycomb-like scaffold in promoting integrated bone-cartilage repair

**FIGURE 7:**
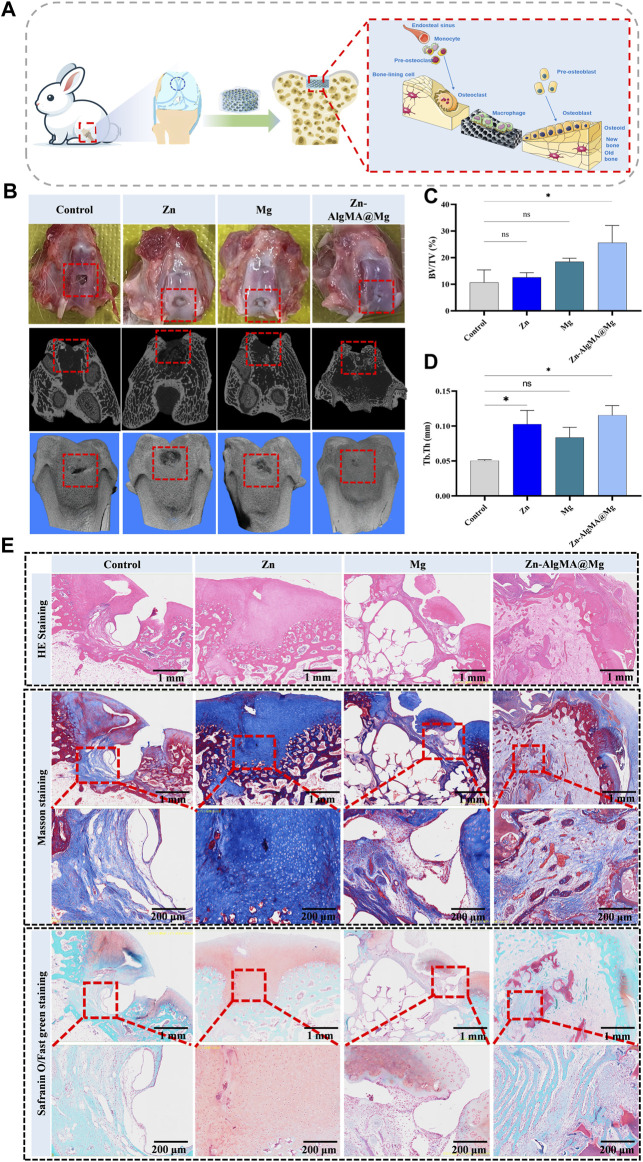
Integrated osteochondral repair effect of Zn-AlgMA@Mg scaffold *in vivo* **(A)** Schematic diagram shows that a Zn-AlgMA@Mg scaffold was implanted into the rabbit femoral intercondylar defect for 10 weeks, and the scaffold played a role in promoting integrated osteochondral repair. **(B)** Visual images, Micro-CT scans and three-dimensional reconstruction images of the defects in each group after repair. **(C)** Comparison of the ratio of new bone volume to total bone volume in each group after repair. **(D)** Comparison of the thickness of new trabecular bone in each group after repair. **(E)** Comparison of histological staining of specimens in each group after repair. Note: *: *p* < 0.05; **: *p* ≤ 0.01; ***: *p* ≤ 0.001; ****: *p* ≤ 0.0001; ns: No significant.

In this experiment, the Zn-AlgMA@Mg scaffold was implanted into the intercondylar defects of rabbit femurs and harvested after 10 weeks to evaluate bone and cartilage regeneration at the defect sites (Figure 7A). Macroscopic observation (Figure 7B) indicated good biocompatibility of this designed Zn-AlgMA@Mg scaffold, with no purulent exudate or inflammatory reactions observed in any joint samples. Apart from the control group, each group exhibited white neotissue in the defect areas, with Zn and Mg groups still showing areas of incomplete repair, whereas the defect area in the Zn-AlgMA@Mg group was completely covered by neotissue. Micro-CT (Figure 7B) revealed that large gaps remained at the center of the defect area in the control group, indicating that spontaneous regeneration by bone marrow mesenchymal stem cells is insufficient for repairing critical-size defects. Conversely, the defect area in the Zn-AlgMA@Mg group was completely filled with neotissue and showed osseous integration with the subchondral bone surrounding the defect. Longitudinal sections showed uncompletely degraded scaffold presence in both Zn and Mg groups, with less neotissue than the Zn-AlgMA@Mg group. Three-dimensional reconstruction (Figure 7B) demonstrated that the tissue surface of the Zn-AlgMA@Mg group post-repair was the most regular and well-integrated with surrounding tissues; this was followed by the Zn and Mg groups, although their surfaces still had irregular tissues.

To further assess the efficiency of integrated bone-cartilage regeneration in different groups, quantitative analysis of the regenerated tissue was conducted. BV/TV results showed that the Zn-AlgMA@Mg group had the largest volume of new bone tissue, significantly different from the control group, followed by the Mg group. The Zn group had the least volume of new bone tissue, with no significant difference compared to the control group (Figure 7C). However, measurements of new trabecular thickness differed slightly; while the Zn-AlgMA@Mg group still had the greatest thickness, the Zn group’s thickness was higher than the Mg group’s, although not significantly (Figure 7D). These findings suggest that both Zn-AlgMA hydrogel and DCPD-Mg alloy stimulate new bone tissue formation, with the performance of the Zn-AlgMA@Mg scaffold surpassing them.

Subsequently, histological staining was performed on femoral intercondylar section samples (Figure 7E). HE staining showed no incidence of osteoarthritis in any samples. Apart from the control and Mg groups, the other two groups had new tissue filling rates exceeding 50% in the defect areas, but the Zn group lacked a clear bone-cartilage interface, filled predominantly with collagen, while the Zn-AlgMA@Mg group had a distinct bone and cartilage boundary, with new bone tissue observed growing along the scaffold’s morphology in the bone defect area. Masson’s trichrome and Safranin-O staining evaluated the scaffold’s effect in newly formed tissue; the control group showed new bone tissue beginning to form at the base of the defect area, growing from inside out, with almost no mature bone tissue visible; the Zn group showed the defect area entirely filled with collagen, with good transition of the cartilage layer to normal tissue, no clear boundary, and many small blood vessels forming at the defect base; the Mg group exhibited a clear bone-cartilage boundary, with many loosely distributed new bone tissues in the bone defect area, still leaving some cavities, possibly related to the rapid degradation rate of magnesium alloy (Liu et al., 2018), and discontinuous cartilage collagen formation at the defect top, yet not fully integrated with normal cartilage tissue; the Zn-AlgMA@Mg group showed a tidemark between subchondral bone and cartilage, abundant new and mature bone tissue in the bone defect area, accompanied by many new blood vessels, and evident collagen deposition in the cartilage area, tightly connected to surrounding normal tissue, with a clear osteogenic area beneath the collagen. This suggests that the Zn-AlgMA@Mg scaffold combines the properties of Zn-AlgMA hydrogel and DCPD-Mg alloy scaffolds, completely replaced by neotissue in the scaffold-filled area, simultaneously promoting bone and cartilage formation, with a clear boundary between cartilage and subchondral bone, and neotissue transitioning well with normal tissue.

In summary, the performance of the Zn-AlgMA@Mg scaffold highlights its potential application in the field of bone-cartilage regenerative materials. Although traditional zinc or magnesium scaffolds can promote the formation of new tissue, their efficiency in bone-cartilage integrated regeneration is limited. In contrast, the biomimetic honeycomb scaffold designed in this experiment combines the advantages of Zn-AlgMA hydrogel and DCPD-Mg alloy. It not only realizes the combination of organic-inorganic matrix to provide a suitable microenvironment for cartilage tissue, but also further reduces the degradation rate of magnesium alloy with the organic polymer coating. It can provide mechanical support for a longer time in the defect and reduce the production of hydrogen and the occurrence of side effects, so as to further improve the biological safety of the scaffold.

On the other hand, because all components of the Zn-AlgMA@Mg scaffold can be safely degraded and absorbed by the human body, it is a biodegradable tissue engineering scaffold on the basis of simulating the natural structure of bone and cartilage tissue, and has multi-directional control performance. It can not only promote the regeneration of bone tissue and cartilage tissue in layers, but also realize seamless integration with surrounding normal tissue. At the same time, it can also play an antibacterial effect, control the occurrence of postoperative infection and reduce complications.

## 4 Disscusion

Currently, clinical treatments for osteochondral defects primarily utilize localized regeneration techniques such as autologous chondrocyte implantation (ACI) (Mistry et al., 2017), microfracture, osteochondral autograft transplantation (OAT) (Richter et al., 2016), and matrix-assisted cartilage repair (MACI) (Wylie et al., 2016). The main objective of these techniques is to restore the cartilage surface and underlying bone by stimulating the body’s repair mechanisms or transplanting healthy cartilage. Although these methods have shown some efficacy in clinical settings, they still present numerous drawbacks, including donor site morbidity, limited integration with surrounding tissues, disease transmission, and repair failures.

In contrast, the biomimetic Zn-AlgMA@Mg scaffold designed in this study offers a novel approach capable of simultaneously achieving cartilage and bone regeneration. Given the substantial differences in the regeneration and repair processes of osteochondral tissues, the porous magnesium alloy scaffold combined with dual-crosslinked sodium alginate hydrogel in this study can respectively mimic the natural extracellular matrix, providing a favorable environment for cell proliferation and differentiation. *In vitro* and *in vivo* results have demonstrated significant osteogenic and chondrogenic properties, with successful integration in a rabbit full-thickness osteochondral defect model, indicating superior efficacy in achieving integrated osteochondral regeneration compared to traditional methods.

During the repair of osteochondral defects, traditional methods such as ACI and OAT involve multiple surgical procedures, increasing the risk of infection and complications. Localized infections are among the most common causes of repair failure. The Zn-AlgMA@Mg scaffold, with its inherent antibacterial properties and controlled degradation performance, ensures a safe and sterile repair environment. The DCPD coating on the magnesium alloy reduces the degradation rate, minimizing the rapid release of hydrogen gas and preventing potential cavitation issues. Additionally, the inclusion of zinc ions in the hydrogel matrix further enhances the scaffold’s antibacterial efficacy, significantly reducing the risk of postoperative infections. Our cytotoxicity and biocompatibility tests confirm the good cellular tolerance of the scaffold components, making it a safer alternative for clinical applications.

Compared to other osteochondral repair scaffolds, the Zn-AlgMA@Mg scaffold exhibits significant advantages in terms of efficacy and tissue integration. Previous studies have indicated that traditional collagen/chondrocyte scaffolds may degrade incompletely *in vivo*, potentially leading to prolonged inflammatory responses (Dong and Lv, 2016). The Zn-AlgMA@Mg scaffold, by incorporating zinc ions, not only enhances the mechanical properties of the hydrogel but also imparts significant antibacterial characteristics, effectively reducing infection risks like honey (Albaridi, 2019). In terms of efficacy, traditional PLGA scaffolds, despite their good biocompatibility, often lack sufficient mechanical strength to provide adequate structural support (Liu et al., 2018). The Zn-AlgMA@Mg scaffold developed in this study combines the superior mechanical properties of porous magnesium alloy with the biocompatibility of dual-crosslinked sodium alginate hydrogel, providing robust mechanical support while promoting both bone and cartilage regeneration. *In vivo* experiments have shown that this scaffold achieves significant tissue integration in a rabbit full-thickness osteochondral defect model, outperforming traditional PLGA scaffolds (Song et al., 2021).

Regarding tissue integration, traditional ceramic-based scaffolds, while effective in bone regeneration, often exhibit poor biodegradability, potentially leading to long-term implant residue (Kamboj et al., 2021). The Zn-AlgMA@Mg scaffold, by incorporating gradually degrading magnesium alloy, facilitates simultaneous bone and cartilage regeneration, thereby avoiding the long-term residue issues associated with ceramic-based scaffolds. Moreover, the release of magnesium and zinc ions during degradation further promotes the formation of new bone and cartilage tissues (Yang et al., 2023).

Therefore, the biomimetic Zn-AlgMA@Mg scaffold presents significant advantages over traditional scaffolds in various aspects and holds remarkable potential for achieving integrated osteochondral repair compared to conventional clinical repair methods. This scaffold promises to provide a novel, effective, and safe solution for the clinical treatment of osteochondral defects.

## 5 Conclusion

Inspired by the honeycomb-like structure in nature, this experiment designed and fabricated a biomimetic honeycomb-like scaffold that possesses antibacterial properties, promotes cell proliferation, and facilitates bidirectional differentiation of stem cells, aimed at integrated repair of osteochondral defects. Previous studies on Zn or Mg-based scaffolds have not explored their application in integrated bone-cartilage defect repair; hence, this experiment is the first to combine organic polymer materials with inorganic metallic materials based on biomimetic principles for use in osteochondral regeneration. The biomimetic scaffold constructed in this research features a honeycomb-like-like metallic scaffold structure and an organic filler that simulates “honey.” It not only demonstrates the advantages of biomimetic structures but also exhibits controlled release properties *in vivo*, along with excellent biocompatibility and antibacterial capabilities. Both *in vivo* and *in vitro* experiments confirmed its ability to promote bidirectional differentiation of BMSCs and facilitate integrated bone-cartilage repair. The scaffold excels not only in the formation of new tissue but also in achieving seamless integration with surrounding normal tissues. Additionally, proteomic analysis further revealed the potential mechanisms underlying the effectiveness of this biomimetic scaffold. In summary, the development of the biomimetic honeycomb-like scaffold in this experiment offers a novel approach for the clinical treatment of osteochondral defects.

## Data Availability

The mass spectrometry proteomics data have been deposited to the ProteomeXchange Consortium (https://proteomecentral.proteomexchange.org) via the iProX partner repository [Ma et al. (2019), Chen et al. (2021)] with the dataset identifier PXD053824: iProX link: https://www.iprox.cn/page/project.html?id=IPX0007755000 ProteomeXchange link: http://proteomecentral.proteomexchange.org/cgi/GetDataset?ID=PXD053824.
