# Supplementary figures and images for: Honeycomb-like biomimetic scaffold by functionalized antibacterial hydrogel and biodegradable porous Mg alloy for osteochondral regeneration
Source: Front Bioeng Biotechnol. 2024 Jul 12;12:1417742. doi: 10.3389/fbioe.2024.1417742 (PMC11273084; doi:10.3389/fbioe.2024.1417742)

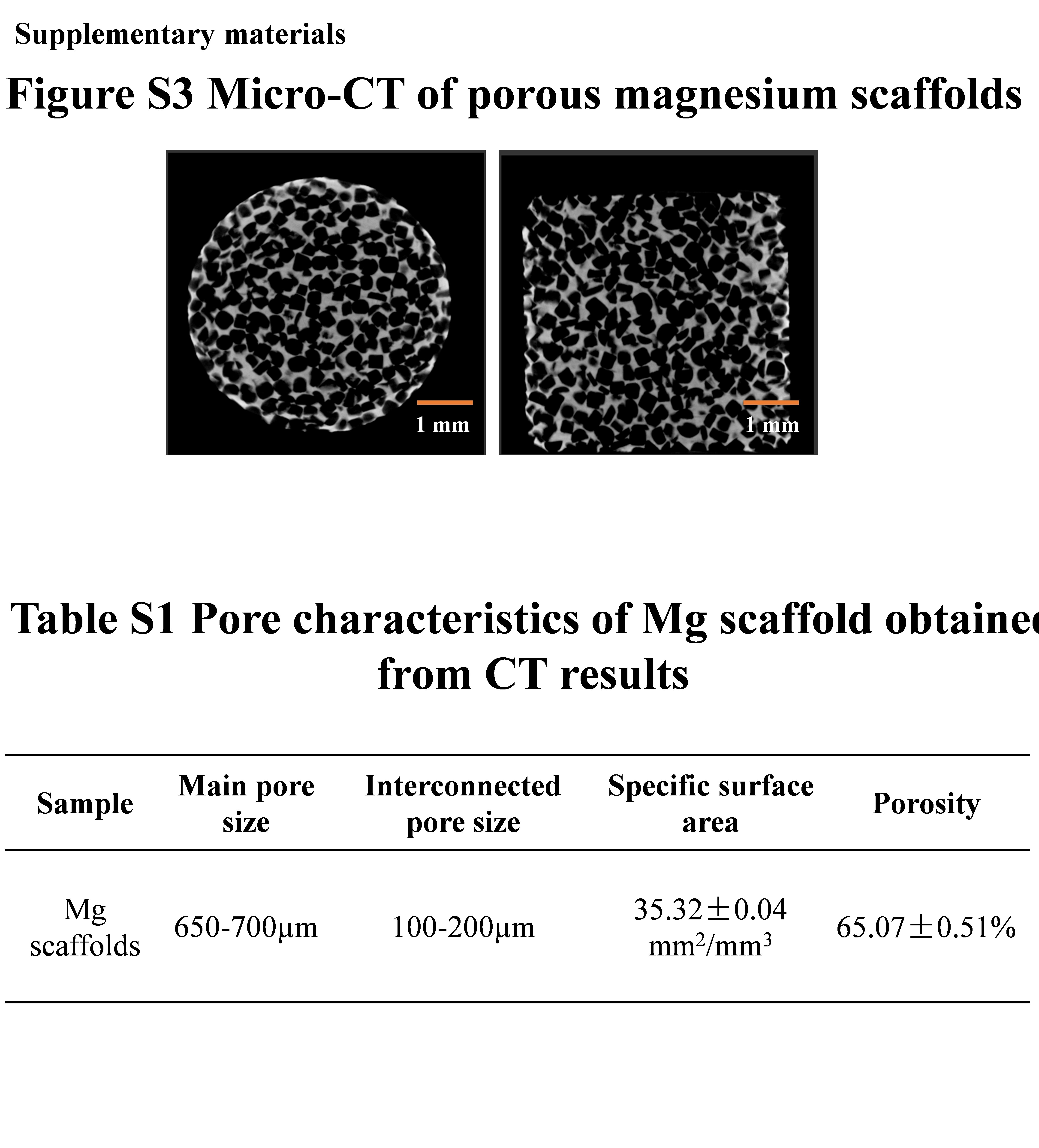

Supplement: Supplementary file 1 [file Image3.JPEG]

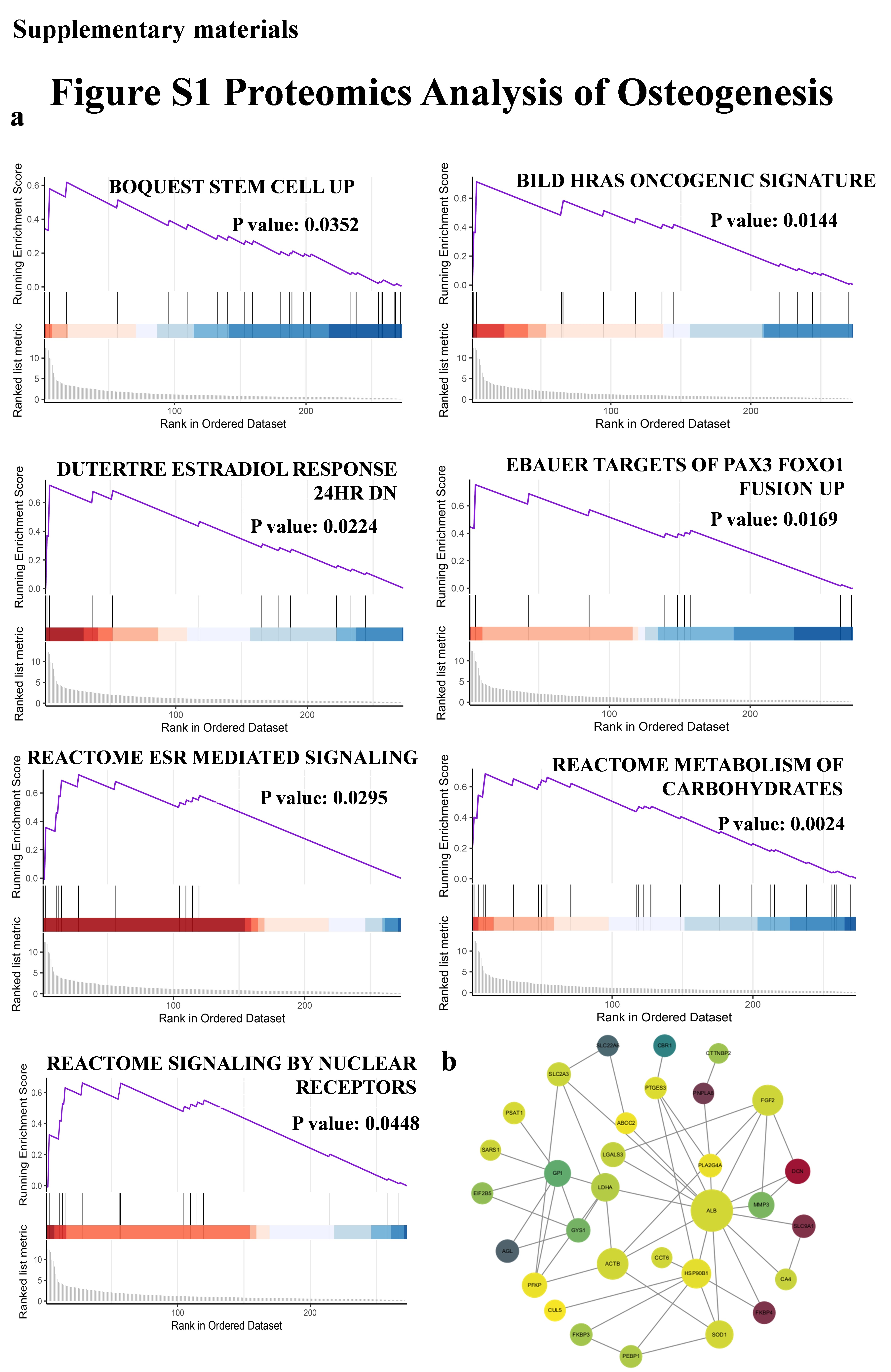

Supplement: Supplementary file 2 [file Image1.JPEG]

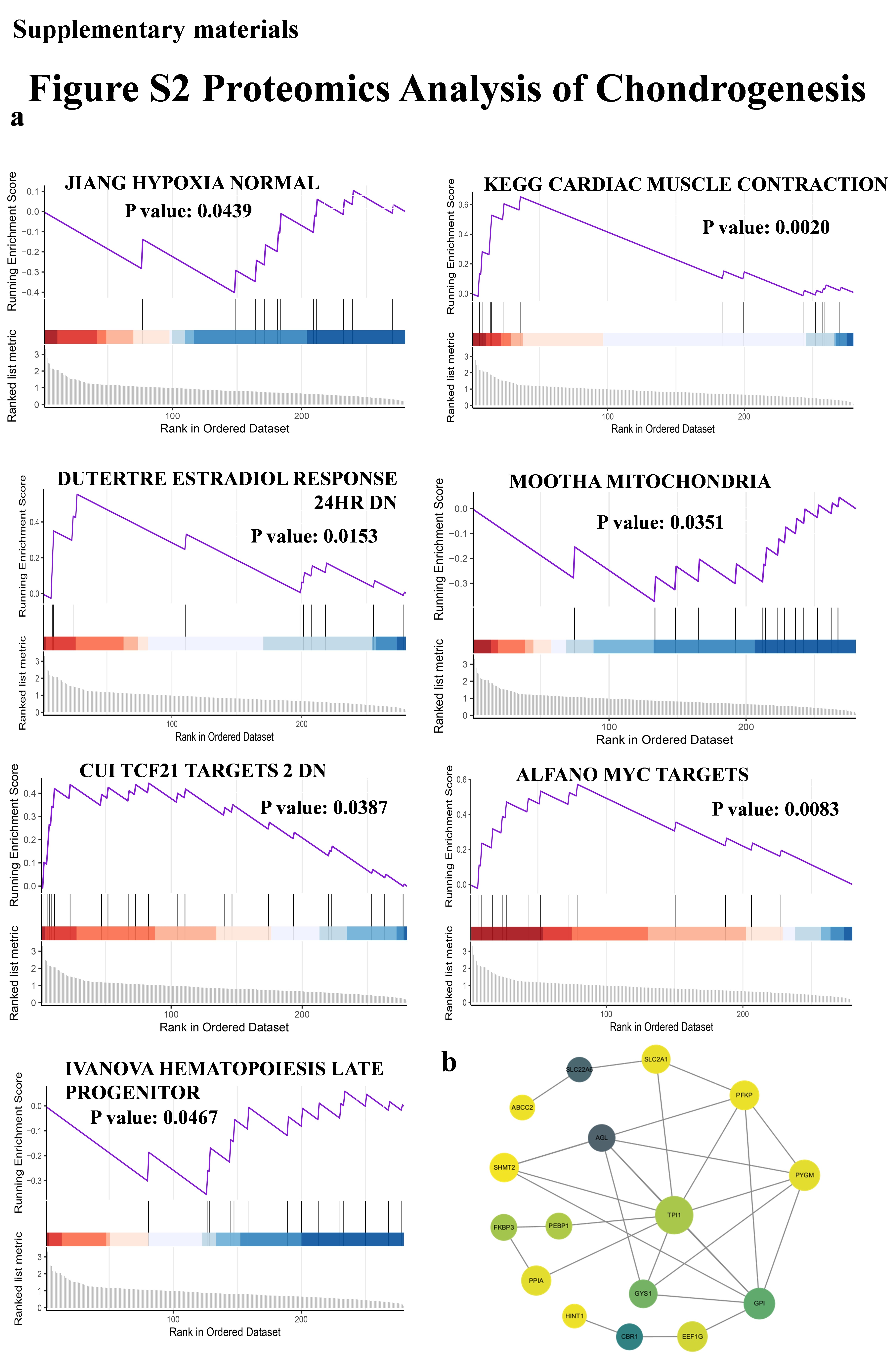

Supplement: Supplementary file 3 [file Image2.JPEG]
